# Highly Sensitive Magnetic-SERS Dual-Function Silica Nanoprobes for Effective On-Site Organic Chemical Detection

**DOI:** 10.3390/nano7060146

**Published:** 2017-06-13

**Authors:** Cheolhwan Jeong, Hyung-Mo Kim, So Yeon Park, Myeong Geun Cha, Sung-Jun Park, San Kyeong, Xuan-Hung Pham, Eunil Hahm, Yuna Ha, Dae Hong Jeong, Bong-Hyun Jun, Yoon-Sik Lee

**Affiliations:** 1 School of Chemical and Biological Engineering, Seoul National University, Seoul 151-742, Korea; purebeam@naver.com (C.J.); sypark1231@naver.com (S.Y.P.); pitpind@naver.com (S.-J.P.); san.volcano@gmail.com (S.K.); 2 Department of Bioscience and Biotechnology, Konkuk University, Seoul 143-701, Korea; hmkim0109@konkuk.ac.kr (H.-M.K.); phamricky@gmail.com (X.-H.P.); greenice@konkuk.ac.kr (E.H.); wes0510@konkuk.ac.kr (Y.H.); 3 Department of Chemistry Education, Seoul National University, Seoul 151-742, Korea; cha6614@snu.ac.kr (M.G.C.); jeongdh@snu.ac.kr (D.H.J.)

**Keywords:** surface-enhanced Raman scattering, magnetic aggregation, on-site detection

## Abstract

We report magnetic silver nanoshells (M-AgNSs) that have both magnetic and SERS properties for SERS-based detection. The M-AgNSs are composed of hundreds of Fe_3_O_4_ nanoparticles for rapid accumulation and bumpy silver shell for sensitive SERS detection by near-infrared laser excitation. The intensity of the SERS signal from the M-AgNSs was strong enough to provide single particle-level detection. We obtained much stronger SERS signal intensity from the aggregated M-AgNSs than from the non-aggregated AgNSs. 4-Fluorothiophenol was detected at concentrations as low as 1 nM, which corresponds to 0.16 ppb. The limit of detection for tetramethylthiuram disulfide was 10 μM, which corresponds to 3 ppm. The M-AgNSs can be used to detect trace amounts of organic molecules using a portable Raman system.

## 1. Introduction

Surface-enhanced Raman scattering (SERS) has been widely utilized as a powerful tool for molecular analysis because of its narrow bandwidth and stability against photobleaching [1,2,3,4,5]. Each organic molecule has a unique SERS spectrum that can act as a molecular fingerprint; thus, a SERS-active material has been used to identify molecules by SERS signals. In this regard, numerous SERS-active materials have been reported to detect various target chemicals, such as pesticides, polycyclic aromatic hydrocarbons, or food contaminants [6,7,8,9]. Among them, novel metal nanoparticles (NPs) are usually exploited for organic chemical detection. The intensity of the SERS signal is highly dependent on the plasmonic property of SERS substrates, which can be greatly influenced by the structure of plasmonic NPs. Thus, proper manipulation of the structure of SERS substrates is needed for increased SERS intensity with greater sensitivities for efficient identification of trace contaminants. One of the important factors that characterize SERS-active NPs is the morphology of the NPs [10]. To obtain different ranges of the absorption spectrum, various shaped nanostructures based on Ag or Au NPs were prepared by conducting galvanic replacement reaction [11,12]. These nanostructures, especially Ag NPs, exhibited specific absorption spectra depending on the concentration of HAuCl_4_. As another nanostructure for effective generation of SERS signal, metal nanoshells attracted lots of interests [13,14]. Metal nanoshells can be excited by a near-infrared (NIR) light source, providing great advantages, such as fewer disturbances from biomolecules which have auto-fluorescence and a deeper tissue penetration capability [15,16]. The bumpy shell structure is more desirable than a smooth one, because the Raman reporter molecules generate much stronger signals when they are located in the nanogaps between the bumps on the surface [17].

Magnetic properties have been widely utilized as a component of complex nanostructures in multifunctional SERS active NPs [18,19,20,21]. They are usually exploited for the separation of cells [22] or small molecules [23] because NP movement is easily controlled by applying an external magnetic field. Such a special modality could be combined with SERS to acquire a synergetic effect for on-site chemical detection. Magnetic-induced accumulation of SERS active NPs is quite effective for increasing SERS intensity that is closely related with detection sensitivity [24]. In order to obtain the magnetic-SERS dual function, NPs which have strong response to an external magnetic field, assembling a large number of magnetic NPs on SERS active NPs rather than using a single magnetic NP is desirable [25].

So far, various approaches have been tried to obtain magnetic-SERS dual functionality. The nanostructures having Fe_3_O_4_ NPs and plasmonic NPs together have been developed by introduction of silica shell or polymer support. However, the nanostructures exhibit weak magnetization due to small numbers of incorporated Fe_3_O_4_ NPs [26,27,28] or were not sufficient to generate strong SERS signals due to insufficient nanogaps between plasmonic NPs [29]. For preparation of metal nanoshell, metal NP seeds were needed beforehand, which is a relatively complex and time-consuming process [30]. Other cases also had some limitations due to polydispersed NPs [31,32], or should be improved for on-site detection with a portable Raman system [33].

**Figure 1:**
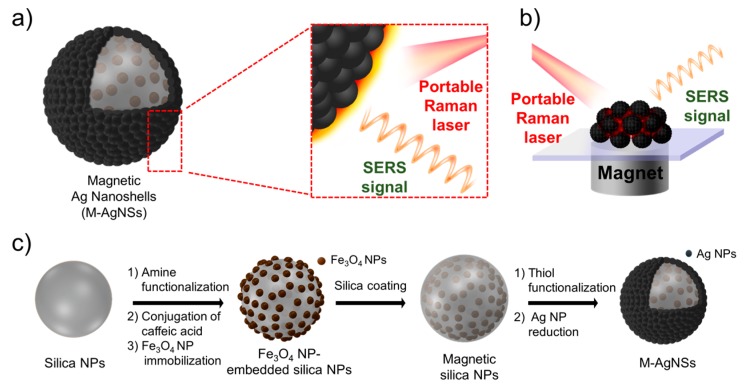
(**a**) Structure of a magnetic silver nanoshell (M-AgNS). The inset shows the enhanced SERS signal that arises from a bumpy M-AgNS surface. (**b**) Magnetic-induced aggregation procedure for sensitive target detection. (**c**) Synthetic scheme for M-AgNSs.

Herein, we have developed multilayered magnetic-SERS dual-function NPs for ultrasensitive, portable, and on-site Raman-based detection of organic chemicals. The magnetic silver nanoshell (M-AgNS) structure was designed to improve the SERS sensitivity. The M-AgNSs are composed of hundreds of Fe_3_O_4_ NPs for rapid accumulation and a bumpy silver shell for sensitive SERS detection using NIR laser excitation, as shown in Figure 1a. Superparamagnetic Fe_3_O_4_ NPs are immobilized on a silica NP, and covered by an additional silica layer. When an external magnetic field is applied, the M-AgNSs show a strong response to the magnetic field because of a large amount of Fe_3_O_4_ NPs assembled on the nanostructure. A silver shell was simply formed on the outer layer of the silica-coated Fe_3_O_4_ NP-embedded silica NP (magnetic silica NP) with seedless single step method. A bumpy silver structure largely contributes to an intrinsically strong SERS signal [17]. The dual functionality of the M-AgNSs can be utilized to generate strong SERS signals after being attracted by a magnet, as shown in Figure 1b.

## 2. Results and Discussion

### 2.1. Preparation and Characterization of M-AgNS

**Figure 2:**
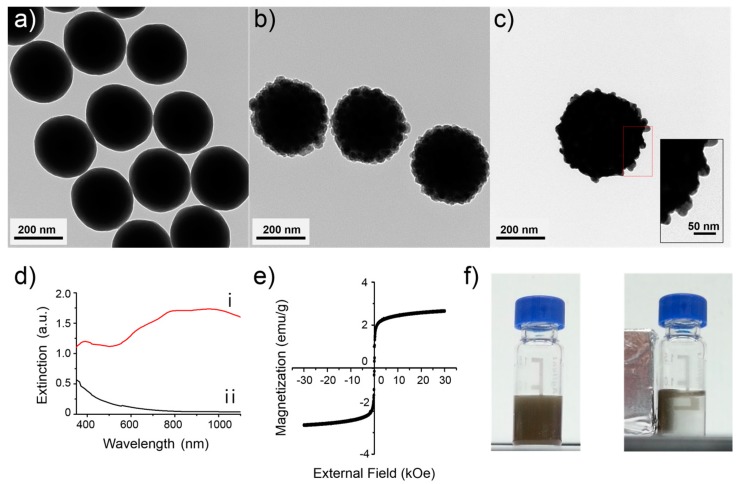
TEM images of (**a**) silica NPs; (**b**) magnetic silica NPs; and (**c**) M-AgNSs; (**d**) Ultraviolet-visible (UV–Vis) absorption spectrum of (**i**) M-AgNSs and (**ii**) magnetic silica NPs; (**e**) Hysteresis loop of M-AgNSs; (**f**) Photographic images of the M-AgNSs before and after being attracted by a magnet for 5 min.

The synthetic procedure of M-AgNS is shown in Figure 1c. First, silica NPs were synthesized using the Stöber method [34] and analyzed by transmission electron microscopy (TEM), as shown in Figure 2a. The silica NPs were spherical and monodispersed (250 ± 10 nm). Catechol groups were exposed on the surface of silica NPs by successively reacting with 3-aminopropyltriethoxysilane (APTS) and caffeic acid. To follow up the surface modification process of silica NPs, zeta potentials of the silica NPs and the amine functionalized silica NPs were measured. Before introduction of amine groups, the zeta potential value was −40 mV (Figure S1). After introduction of amine groups, the zeta potential value was shifted toward positive direction (−13 mV). This result indicates that amine groups were introduced to the surface of silica NPs, although not completely replacing pre-existed hydroxyl groups.

Fe_3_O_4_ NPs could be immobilized on the catechol functionalized silica NPs by the strong interaction between catechol groups and Fe_3_O_4_ NPs [35,36]. Oleate stabilized Fe_3_O_4_ NPs were modified with polyvinylpyrrolidone (PVP, MW 10,000), to react with catechol functionalized silica NPs in amphiphilic solvent. Then, additional silica encapsulation was conducted to prevent detaching of the Fe_3_O_4_ NPs from the silica NPs. Approximately 400 units of Fe_3_O_4_ NPs were assembled on each silica NP, and the thickness of the silica layer on the outer part of the magnetic silica NPs was approximately 10 nm, as shown in Figure 2b. Then, thiol groups were introduced onto the magnetic silica NPs by treating with 3-mercaptopropyltrimethoxysilane (MPTS). Silver ions were effectively reduced on the surface of the magnetic silica NPs because of the interaction between the thiol groups of MPTS and the silver ions, producing silver NPs and ultimately leading to the silver shell [37]. Addition of PVP (MW 40,000) during silver reduction process is needed to prevent as-synthesized silver NPs from aggregation [38]. Octylamine and ethylene glycol have important roles in the nucleation and growth of silver NPs on the surface of the magnetic silica NPs [39]. The resulting M-AgNSs have a bumpy shell structure, as shown in Figure 2c.

The optical properties of the M-AgNSs were characterized by UV–Vis spectroscopy (Figure 2d). The M-AgNSs showed a broad extinction band in the NIR region (approximately 600–1100 nm) as shown in Figure 2d (i), while the magnetic silica NPs as a control, showed no characteristic feature of plasmon extinction in the NIR region as shown in Figure 2d (ii). Metal nanoshells are known to have NIR active property, because of their different plasmonic property from simple spherical metal NPs [40,41,42]. The extinction spectrum of M-AgNSs indicates that Ag^+^ ions were completely reduced to form shell structure.

Magnetic property of NPs can be characterized by the saturation magnetization value. We confirmed the magnetic property of the M-AgNSs by analyzing the field dependent magnetization at 300 K. The saturation magnetization of the M-AgNSs was 2.7 emu/g, and there was no remanence magnetization when the external magnetic field was removed, as shown in the magnetization curve (Figure 2e). The non-remanence magnetization is attributed to the individual Fe_3_O_4_ NP (18 nm), which are small enough to maintain superparamagnetic property [43]. This can explain the observed superparamagnetic behavior of the M-AgNSs. Meanwhile, the saturation magnetization of M-AgNSs can be interpreted in terms of the number of individual Fe_3_O_4_ NPs and the total mass of M-AgNS. Approximately 400 Fe_3_O_4_ NPs are responding to the external magnetic field and the mass of M-AgNS is approximately ca. 8000 times bigger than individual Fe_3_O_4_ NP (size of M-AgNS and Fe_3_O_4_ NP is 400 nm and 18 nm, respectively). Simple estimation reveals that the saturation magnetization value of M-AgNS is 0.05 times lower than that of individual Fe_3_O_4_ NP, ca. 3 emu/g. In fact, the observed value of M-AgNS is 2.7 emu/g, which is similar to the estimated value. Overall, M-AgNS has 400 times stronger response than individual Fe_3_O_4_ NPs, maintaining superparamagnetic property, and thus, M-AgNSs were strongly attracted by a magnet within 5 min (Figure 2f).

### 2.2. SERS Property of M-AgNSs

**Figure 3:**
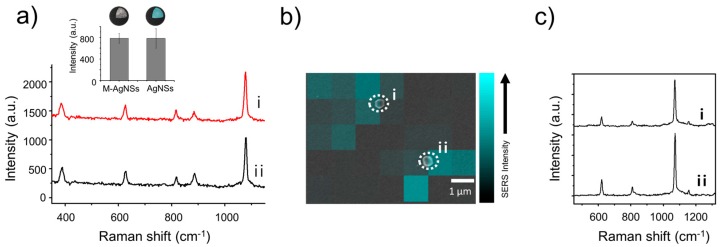
(**a**) SERS spectra of 4-FBT on (**i**) M-AgNSs and (**ii**) AgNSs. The inset shows the intensities of the 1075 cm^−1^ peak of 4-FBT. The spectra were obtained using a portable Raman system with a 785 nm laser at a power of 30 mW for 1 s. (**b**) SERS intensity map of 4-FBT-treated M-AgNSs. The corresponding SEM image was overlaid with the SERS map. (**c**) SERS spectra obtained from single M-AgNS particles. Each spectrum (**i**,**ii**) corresponds to the single particles in SERS intensity map.

The SERS signal from the M-AgNSs was obtained using a portable Raman system with 785 nm laser irradiation (Figure 3a). 4-Fluorobenzenethiol (4-FBT), a Raman reporter molecule, was incubated with the M-AgNSs in ethanol. The thiol group of 4-FBT can interact strongly with the silver shell; thus, a SERS signal could be obtained from 4-FBT absorbed on the M-AgNSs. The SERS intensity from the M-AgNSs was compared to that from the AgNSs that have a similar structure as M-AgNSs but without the Fe_3_O_4_ NPs. The AgNSs were prepared by forming a silver layer on silica NPs instead of magnetic silica NPs. The SERS intensities were determined by measuring the height of the 1075 cm^−1^ peak (the highest peak in the spectrum of 4-FBT) in the SERS spectrum; the difference between the SERS intensities of the M-AgNSs and AgNSs was not significant. This result suggests that the existence of Fe_3_O_4_ NPs at the inner part of the M-AgNSs did not significantly affect the intensity of the SERS signal. Furthermore, we collected the SERS signal from a single M-AgNS particle. A drop of the M-AgNS solution (0.5 mg/mL in ethanol) was placed on a patterned slide glass to measure the SERS signal. The SERS signal was collected using point-by-point mapping with a 1 μm step size using a 660 nm laser at a power of 2 mW with an exposure time of 1 s per point. Then, a scanning electron microscope (SEM) image was obtained in the same area in which the SERS mapping was performed. The location of each M-AgNS particle was aligned exactly with the SERS map. As shown in Figure 3b,c, the SERS signal intensity from the M-AgNSs was strong enough to be detected at the single particle-level (SERS enhancement factor: 1.4 × 10^6^). This sensitive SERS property originated from the shell structure of M-AgNSs. To investigate the effect of Ag NP’s density on the SERS intensity of M-AgNSs, weight ratio of AgNO_3_ to core silica NPs (AgNO_3_/silica NPs) was varied during formation of Ag shell layer. As the ratio of AgNO_3_ to core silica NPs decreased, the extinction of M-AgNSs gradually decreased in the NIR region while their extinction around 400 nm maintained, which is characteristic feature of small sized Ag NPs (Figure S2b). This reflects that with decreasing the amount Ag^+^ ion per core silica NPs, Ag shell structure is broken and Ag NPs are formed apart from one another, causing decreased extinction in the NIR region. These results reveal that above a certain amount of Ag^+^ ion is necessary for the formation of bumpy shell structure having strong NIR extinction and sensitive SERS property in NIR region (Figure S2a) [44].

### 2.3. Magnetic-Induced Aggregation of M-AgNSs for Sensitive SERS Detection

**Figure 4:**
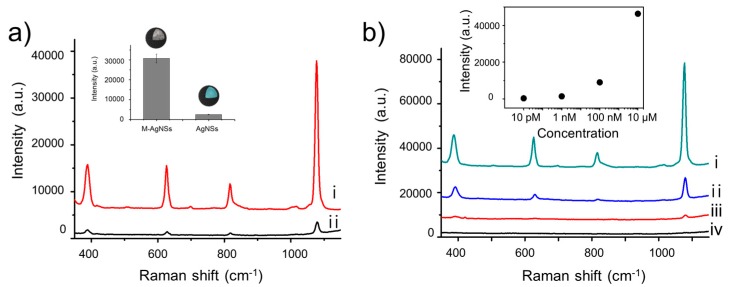
(**a**) SERS spectra of 4-FBT on (**i**) M-AgNSs and (**ii**) AgNSs after being attracted by a magnet. The inset shows the intensities for the 1075 cm^−1^ peak of 4-FBT. The spectra were obtained using a portable Raman system with a 785 nm laser at a power of 3 mW for 1 s. (**b**) SERS spectra of 4-FBT on M-AgNSs. The concentrations of 4-FBT were (**i**) 10 μM, (**ii**) 100 nM, (**iii**) 1 nM, and (**iv**) 10 pM. The inset shows the intensities of the 1075 cm^−1^ peak of 4-FBT. The spectra were obtained using a 785 nm laser at a power of 30 mW for 1 s.

Several factors are related with the enhanced SERS intensity when NPs are accumulated by external magnetic field. Generation of resonant interaction between plasmonic NPs in close proximity, so called “hot spot”, can contribute to the enhancement of Raman signal [10]. The number of hot spots as well as the degree of enhancement from each hot spot should be considered simultaneously to explain the signal enhancement by hot spots [45,46]. In addition, as the number of NPs within incident laser spot increases, Raman intensity will increase proportionally due to the increase in the number of signal generating molecules [47,48]. When the M-AgNSs were aggregated into specific point by external magnetic force, hot spots are generated between M-AgNSs in contact or close proximity and the number of hot spots can be increased as the number density of M-AgNSs increases by aggregation. The increased number density of M-AgNSs also enhanced the Raman signal generating molecules, consequently contributing to the overall increase of Raman intensity. To evaluate the effectiveness of magnetic-induced aggregation, we investigated the SERS signal of the M-AgNSs after being attracted by a magnet. The 4-FBT-treated M-AgNSs were re-dispersed in water, and a drop of the solution (0.5 mg/mL) was placed on a glass slide. Then, we placed a magnet underneath the glass slide, as shown in Figure 1b. After 10 min, the M-AgNSs were completely accumulated around the magnet. In contrast, when a drop of the AgNS solution was placed on a glass slide in the same manner, the magnet did not collect the AgNSs because they have no magnetic property. As shown in Figure 4a, the SERS intensity of the aggregated M-AgNSs is 12 times stronger than that of the non-aggregated AgNSs, showing the strong enhancement of the SERS signal by magnetic-induced aggregation. Different concentrations of 4-FBT solutions were treated with M-AgNSs to investigate the limit of detection (LOD) by this method. As shown in Figure 4b, the intensity of the SERS signal decreased as the concentration of 4-FBT decreased. Finally, the SERS signal of 4-FBT could be obtained at a concentration as low as 1 nM, which corresponds to 0.16 ppb.

### 2.4. Thiram Detection Using M-AgNSs

Furthermore, we treated tetramethylthiuram disulfide (thiram) solution with M-AgNSs to investigate its ability to detect a trace amount of the pesticide. A drop of the thiram-treated M-AgNSs solution was placed on a glass slide and attracted by a magnet for 10 min. The SERS signal from the thiram molecules on the M-AgNSs could be obtained using a portable Raman system. The LOD for thiram was 10 μM, which corresponds to 3 ppm (Figure S3).

## 3. Materials and Methods

### 3.1. Materials

All reagents are used without further purification process. Oleate-stabilized Fe_3_O_4_ nanoparticles (NPs) were purchased from Ocean Nanotech (Springdale, AR, USA). Absolute ethanol was purchased from Carlo Erba. Tetraethyl orthosilicate (TEOS), ammonium hydroxide (NH_4_OH, 28 wt % in water), 3-aminopropyltriethoxysilane (APTS), caffeic acid, 3-mercaptopropyltrimethoxysilane (MPTS), silver nitrate (AgNO_3_), ethylene glycol, polyvinylpyrrolidone (PVP, MW 10,000 and 40,000), octylamine and 4-FBT (4-fluorobenzenethiol) were purchased from Sigma Aldrich (St. Louis, MO, USA). 2-(1H-Benzotriazole-1-yl)-1,1,3,3-tetramethyluronium hexafluoro–phosphate (HBTU) and hydroxybenzotriazole (HOBt) were purchased from Bead Tech (Ansan, Korea). Tetramethylthiuram disulfide (thiram) was purchased from Alfa Aesar (Ward Hill, MA, USA). Ethanol (95%), *N*,*N*-dimethylformamide (DMF), methylene chloride (MC), diethyl ether, and *N*,*N*-diisopropylethylamine (DIEA) were purchased from Daejung Chemical (Siheung, Korea).

### 3.2. Synthesis of Magnetic Silica NPs

Silica NPs were synthesized according to the well-known Stöber method. TEOS (1.6 mL) and NH_4_OH (4 mL) were added to absolute ethanol (40 mL, 99.5%) and stirred for 18 h at room temperature. The resulting silica NPs were centrifuged (7000 rpm, 15 min) and washed with ethanol several times. To introduce amine functional groups, silica NPs (40 mg) dispersed in ethanol (20 mL) were reacted with APTS (100 μL) and NH_4_OH (100 μL) for 18 h. The reaction mixture was centrifuged (7000 rpm, 15 min) and washed with DMF several times. The resulting amine functionalized silica NPs (20 mg) were dispersed in DMF (5 mL), and reacted with caffeic acid (7.2 mg) and same equivalent of HBTU, HOBt, and DIEA for 3 h. The resulting catechol-functionalized silica NPs were centrifuged (7000 rpm, 15 min) and washed with DMF. Meanwhile, in order to stabilize Fe_3_O_4_ NPs with PVP (MW 10,000), oleate-stabilized Fe_3_O_4_ NPs (2.5 mg dispersed in 100 μL chloroform) were poured into DMF/MC co-solvent (1:1 v/v, 5 mL) and PVP (MW 10,000, 120 mg) was added. The mixture was sonicated and heated to 100 °C for 3 h, and cooled. The PVP-stabilized Fe_3_O_4_ NPs were slowly transferred into diethyl ether (10 mL). The solution was centrifuged (4500 rpm, 5 min) and re-dispersed in ethanol. The PVP-stabilized Fe_3_O_4_ NPs (0.08 mg/mL in 5 mL ethanol) and catechol-functionalized silica NPs (0.2 mg/mL in 5 mL DMF) were mixed and sonicated for 1 h. The mixture was centrifuged (6000 rpm, 10 min) and washed with ethanol. For silica coating, TEOS (50 μL) and NH_4_OH (100 μL) were added to the Fe_3_O_4_ NPs-embedded silica NPs (dispersed in 5 mL ethanol) and reacted for 18 h. The resulting magnetic silica NPs were centrifuged (6000 rpm, 10 min) and washed several times with ethanol.

### 3.3. Synthesis of M-AgNSs and AgNSs

To obtain thiolated magnetic silica NPs, MPTS (10 μL) and NH_4_OH (50 μL) were added to the magnetic silica NPs suspension (1 mg/mL in ethanol). The mixture was shaken for 1 h at 50 °C. The resulting thiolated magnetic silica NPs were centrifuged (13,000 rpm, 5 min) and washed several times with ethanol to remove excess reagent. Then, the thiolated magnetic silica NPs were dispersed in ethanol (100 μL). For the introduction of Ag shell, thiolated magnetic silica NPs (10 mg/mL in 50 μL ethanol) were added to PVP solution (MW 40,000, 0.2 mg in 1 mL ethylene glycol), followed by mixing with ethylene glycol solution (1 mL) that contains AgNO_3_. The final concentration of AgNO_3_ was 7.3 mM. Then octylamine (4 μL) was poured quickly into the solution and the mixture was stirred for 1 h at 25 °C. The final product was centrifuged (13,000 rpm, 5 min) and washed several times with ethanol. Meanwhile, AgNSs were prepared by using silica NPs solution (1 mg/mL in ethanol) instead of magnetic silica NPs solution, and the following process was identical to the synthesis of M-AgNSs. The SERS spectra from the M-AgNSs and AgNSs were obtained using a portable Raman system with a 785 nm laser at a power of 30 mW for 1 s. When different weight ratio of AgNO_3_ to silica NPs was applied to the formation of Ag shell, the amount of thiolated magnetic silica NPs was adjusted. That is, 10, 20, 30, 40, 50 μL of thiolated magnetic silica NPs (50 mg/mL in EtOH) were reacted with AgNO_3_ and octylamine. The SERS spectra from the M-AgNSs and AgNSs were obtained using a portable Raman system with a 785 nm laser at a power of 60 mW for 1 s.

### 3.4. Characterization of NPs

Transmission electron microscopic (TEM) images were obtained by a LIBRA 120 (Carl Zeiss, Oberkochen, Germany). Zeta potential of NPs were analyzed with zeta-sizer (Nano-ZS, Malvern, UK). UV-Vis extinction spectra were obtained with an Optigen 2120UV (Macasys, Daejeon, Korea). Field-dependent magnetization was analyzed with PPMS-14 (Quantum Design, San Diego, CA, USA). The SERS spectra were obtained with a portable Raman system (i-Raman, B&W TEK, Newark, DE, USA). SERS mapping was proceeded with a micro-Raman system (LabRam 300, JY-Horiba, Edison, NJ, USA). SEM images were obtained with SUPRA 55VP (Carl Zeiss, Oberkochen, Germany).

### 3.5. Single Particle SERS Measurement

M-AgNSs (0.5 mg) were added to 4-FBT solution (1 mM in ethanol) and reacted for 1 h. Then, the mixture was centrifuged (13,000 rpm, 5 min) and washed several times with deionized (DI) water. A drop (10 μL) of M-AgNSs solution (0.5 mg/mL in 1 mL DI water) was put on a patterned slide glass. After obtaining point-by-point SERS mapping image with a 1-μm step size for 1 s using a 660 nm laser line, the corresponding region was investigated by SEM image. The spectra were obtained using a micro Raman system with a 660 nm laser at a power of 2 mW for 1 s.

### 3.6. Calculation of the SERS Enhancement Factor

SERS enhancement factor (EF) for a 4-FBT-treated M-AgNS was estimated using the following equation: EF = (I_SERS_/N_SERS_)/(I_normal_/N_normal_), where *I*_SERS_ and I_normal_ are the intensity of the bands from SERS and normal Raman scattering, respectively, and N_normal_ and N_SERS_ are the number of Raman reporter molecules (4-FBT) in pure form and self-assembled one on the surface of silver NP in M-AgNSs. To estimate EF, the representative band at 1075 cm^−1^ of 4-FBT was used. The Raman signals from both single M-AgNS particle and neat 4-FBT were measured in identical condition with 660 nm photo-excitation, 30 mW laser power, 1 s acquisition time, using the same light collection lens. The scattering volume in normal Raman measurements was estimated to be 18.8 μm^3^ as a cylinder form with a diameter of 2 μm and a height of 1200 μm. Since the molecular weight and density of 4-FBT are 125.19 g/mol and 1.203 g/cm^3^, respectively, the *N*_normal_ was estimated to be 6.2 × 10^10^. The N_SERS_ was calculated by geometrically estimating the particle’s surface area and a molecular footprint of 4-FBT (0.383 nm^2^/mol) assuming that the Raman reporter molecules were adsorbed on the surface of NP as a monolayer with 100% coverage [49]. The N_SERS_ from 10 M-AgNSs, which are included cylindrical form, was estimated as 5.03 × 10^6^. Thus, EF for a 4-FBT-treated M-AgNS was estimated as 1.4 × 10^6^.

### 3.7. Magnetic-Induced Aggregation of M-AgNSs

For obtaining SERS signal from 4-FBT, M-AgNSs (0.5 mg) were dispersed in ethanol containing 4-FBT, and incubated for 1 h. The mixture was centrifuged (13,000 rpm, 5 min) and washed several times with DI water. A drop (10 μL) of M-AgNSs solution (0.5 mg/mL in 1 mL DI water) was transferred to a glass slide. Then a magnet was located underneath the glass slide, and M-AgNSs were accumulated by the magnet for 10 min. The spectra were obtained using a portable Raman system with a 785 nm laser at a power of 3 mW for 1 s.

For obtaining SERS signal from thiram, M-AgNSs were dispersed in ethanol containing thiram, and reacted for 1 h. Then, a drop (2 μL) of M-AgNSs solution (0.5 mg/mL in 1 mL ethanol) was transferred to a glass slide. After a magnet was located underneath the glass slide, the NPs were accumulated for 10 min. The spectra were obtained using a portable Raman system with a 785 nm laser at a power of 60 mW for 5 s.

## 4. Conclusions

We have fabricated M-AgNSs, which has both magnetic and SERS properties for sensitive detection of chemicals. The M-AgNSs contain hundreds of Fe_3_O_4_ NPs and have a bumpy silver shell. Thus, a strong SERS signal could be obtained from the absorbed target because of the bumpy structure and magnetic-induced aggregation. The sensitivity of this method was confirmed by obtaining the SERS signal from a trace amount of molecules in a solution with a portable Raman system. Furthermore, rapid accumulation of the M-AgNSs caused by the strong magnetic response provides great advantages for on-site detection of contaminants. Based on the magnetic-SERS dual functionality, M-AgNSs are expected to be utilized as a practical and effective nanoprobe for on-site and highly-sensitive detection of contaminants.

## Supplementary Materials

The following are available online at http://www.mdpi.com/2079-4991/7/6/146/s1, Figure S1: The zeta potential values of silica NPs and amine-functionalized silica NPs, Figure S2: (a) SERS intensity of M-AgNSs treated with different weight ratio of AgNO_3_ to core silica NPs (AgNO_3_/ silica NP) during Ag^+^ ion reduction process. The SERS intensities of NPs were analyzed at 1075 cm^−1^ peak from the spectra, which were obtained using a portable Raman system with a 785 nm laser at a power of 60 mW for 1 s. (b) UV–Vis absorption spectrum of M-AgNSs treated with different weight ratio of (AgNO_3_/silica NP) (i) 5, (ii) 2.5, (iii) 1.6, (iv) 1.25 and (v) 1 during Ag^+^ ion reduction process, Figure S3: SERS spectra of (i) thiram-treated M-AgNSs and (ii) non-treated M-AgNSs after being accumulated by using a magnet. The concentration of thiram was 10 μM. The spectra were obtained using a portable Raman system (785 nm laser; 60 mW of power for 5 s).
