# Supplementary material for: Highly Sensitive Magnetic-SERS Dual-Function Silica Nanoprobes for Effective On-Site Organic Chemical Detection
Source: Nanomaterials (Basel). 2017 Jun 13;7(6):146. doi: 10.3390/nano7060146 (PMC5485793; doi:10.3390/nano7060146)
Supplement: Supplementary file 1 [file nanomaterials-07-00146-s001.pdf]

Supplementary material

Highly Sensitive Magnetic-SERS Dual-Function Silica Nanoprobes for Effective On-Site Organic Chemical Detection

Cheolhwan Jeong,[1,†] Hyung-Mo Kim,[2,†] So Yeon Park,[1] Myeong Geun Cha,[3] Sung-Jun Park,[1] San Kyeong,[1] Xuan-Hung Pham,[2] Eunil Hahm,[2] Yuna Ha,[2] Dae Hong Jeong,[3] Bong-Hyun Jun[2,*] and Yoon-Sik Lee[1,*]

[1] School of Chemical and Biological Engineering, Seoul National University Seoul 151-742, Republic of Korea; purebeam@naver.com (C.J.); sypark1231@naver.com (S.Y.P.); pitpind@naver.com (S -J.P.); san.volcano@gmail.com (S.K.);

[2] Department of Bioscience and Biotechnology, Konkuk University, Seoul 143-701, Republic of Korea; hmkim0109@konkuk.ac.kr (H.-M.K.); phamricky@gmail.com (X.-H.P.); greenice@konkuk.ac.kr (E.H.); wes0510@konkuk.ac.kr (Y.H.);

[3] Department of Chemistry Education, Seoul National University, Seoul 151-742, Republic of Korea; cha6614@snu.ac.kr (M. G. C.); jeongdh@snu.ac.kr (D. H. J.)

* Correspondence: bjun@konkuk.ac.kr (B.-H.J.); yslee@snu.ac.kr (Y.-S.L); Tel.: +82-2-450-0521 (B.-H.J.); Tel.: +82-2-880-7073 (Y.-S.L.)

† These authors contribute equally to this work.

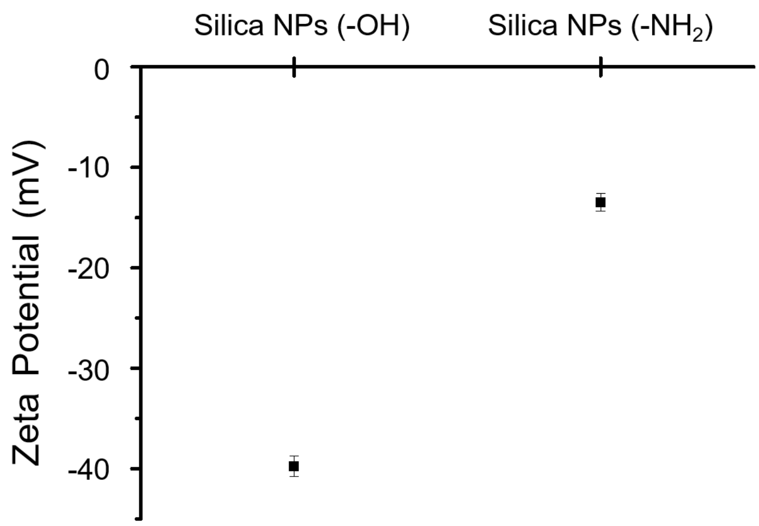

Figure S1. The zeta potential values of silica NPs and amine-functionalized silica NPs.

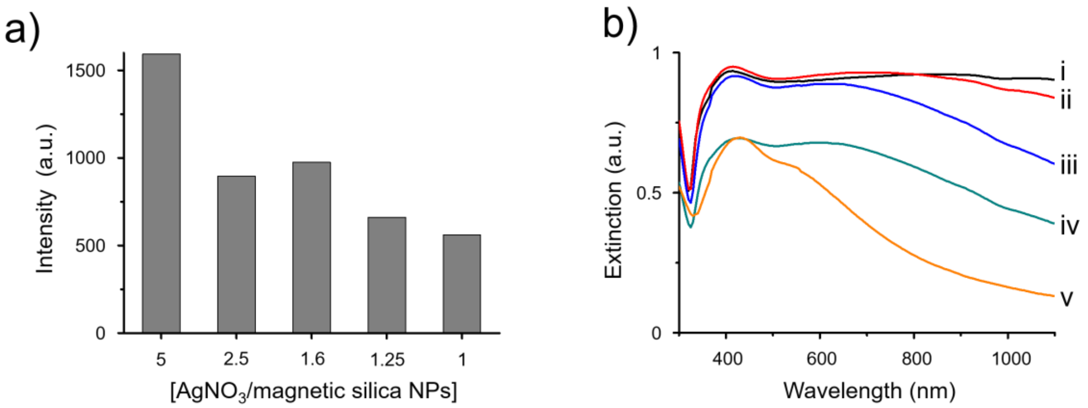

Figure S2. (a) SERS intensity of M-AgNSs treated with different weight ratio of $AgNO_3$ to core silica NPs [$AgNO_3$/Silica NP] during Ag^+ ion reduction process. The SERS intensities of NPs were analyzed at 1075 cm^{-1} peak from the spectra, which were obtained using a portable Raman system with a 785 nm laser at a power of 60 mW for 1 s. (b) UV-Vis absorption spectrum of M-AgNSs treated with different weight ratio of [$AgNO_3$/silica NP] i) 5, ii) 2.5, iii) 1.6, iv) 1.25 and v) 1 during Ag^+ ion reduction process.

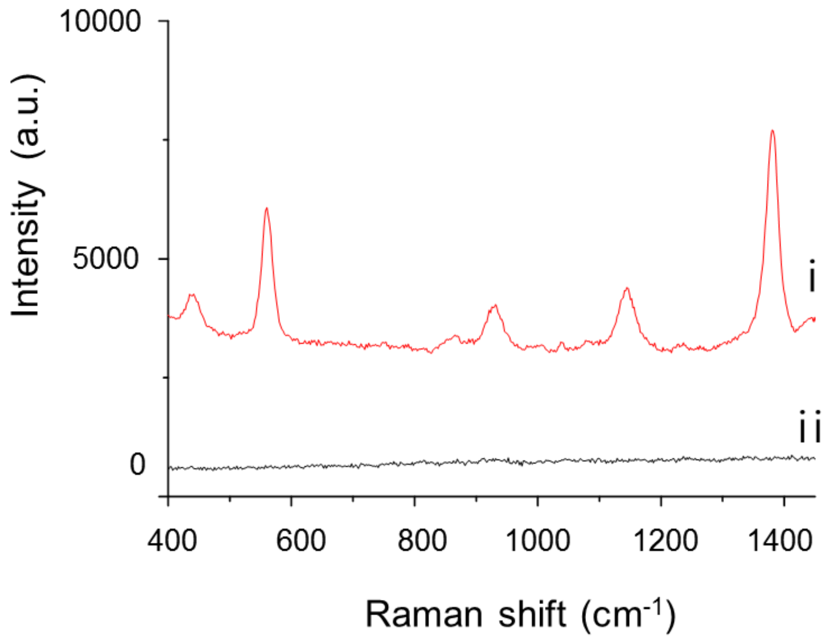

Figure S3. SERS spectra of i) thiram-treated M AgNSs and ii) non-treated M AgNSs after being accumulated by using a magnet. The concentration of thiram was 10 μM. The spectra were obtained using a portable Raman system (785 nm laser; 60 mW of power for 5 s).
